# Closure of a large myelomeningocele defect using the V–Y rotation advancement flap (butterfly flap): a case report and literature review

**DOI:** 10.1080/23320885.2021.1971528

**Published:** 2021-08-31

**Authors:** Hatan Mortada, Tareg Alhablany, Tanveer A. Bhat, Abdulla Al Tamimi

**Affiliations:** a Division of Plastic Surgery, Department of Surgery, King Saud University Medical City, King Saud UniversityRiyadh, Saudi Arabia; b Department of Plastic Surgery and Burn Unit, King Saud Medical City, Riyadh, Saudi Arabia; c Faculty of Medicine, King Abdulaziz University, Jeddah, Saudi Arabia

**Keywords:** Meningomyelocele, butterfly flap, advancement flap, surgical technique, reconstructive surgery

## Abstract

Early coverage of myelomeningocele (MMC) defects within the post-delivery period is crucial for decreasing mortality rates. Herein, we report the case of a premature 5-day-old male neonate with large MMC defect successfully managed using a quadruple V-Y rotation advancement flap (butterfly technique), an effective surgical technique for large MCC defects.

## Introduction

Myelomeningocele (MMC) is the most common cause of open spinal dysraphism among newborns [1] and commonly occurs in the lumbosacral region, with an overall incidence of 1 in 1,000 newborns [2,3]. Morbidities associated with MMC include sensory and lower limb motor deficits and bladder and rectal dysfunctions. The mortality rate in untreated cases reaches 65% by the age of 6 months [4]. Early coverage of MMC defects within the post-delivery period is crucial to reduce mortality rates by protecting the incompletely closed spinal cord from central nervous system infections and preventing a cerebrospinal fluid (CSF) leak [5]. Most MMC defects can be covered and closed primarily. However, direct closure is impossible in 25% of cases [6]. Large MMC reconstruction represents a challenge and requires expert reconstructive surgeons [1]. Various surgical options have been reported for MMC closure, including skin flaps, skin grafts, fasciocutaneous flaps, muscle flaps, and musculocutaneous flaps. Herein, we report the use of a quadruple V–Y rotation advancement flap (butterfly technique) to cover the wound of a large MMC defect in a preterm baby. We also present a brief review of the relevant literature on a large MMC coverage.

## Case presentation

### Case history

**Figure 1.**
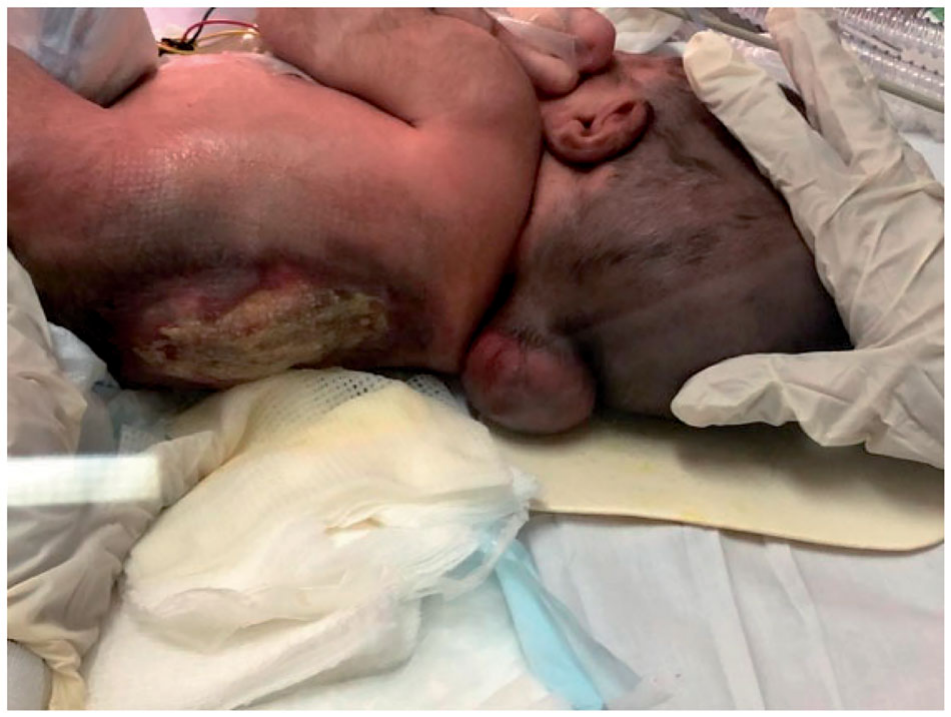
A large thoracolumbar meningomyelocele in a newborn.

A 5-day-old male neonate was delivered at 35 weeks of gestation *via* cesarean section at our institute, King Saud Medical City in Riyadh, Saudi Arabia, who had several congenital defects involving skeletal dysplasia, hydrocephalus, meningoencephalocele, and thoracolumbar MMC at the T6–L2 level (Figure 1). Because of respiratory distress in the postnatal period, the infant was on invasive mechanical ventilation in neonatal intensive care unit (NICU) settings. During evaluation, the patient weighed 2.9 kg with a head circumference of 28.5 cm and Apgar score of 9/9. The baby had hemiplegia. There were two MMCs, one non-ruptured in the cervical region of size 8 × 8 cm^2^, and the other in the thoracolumbar area of size 12 × 16 cm^2^ ruptured with a ratio of its maximum width to that of back >0.5, with a leaking CSF. Moreover, the thoracolumbar spine was kyphotic. No urogenital abnormalities were observed. In the prenatal period, the baby’s mother had two previous miscarriage histories of unidentified causes and recurrent episodes of lower urinary tract infections treated with nitrofurantoin during pregnancy. Signed consent to use preoperative and postoperative images for publication was obtained from the parents.

## Surgical technique

**Figure 2.**
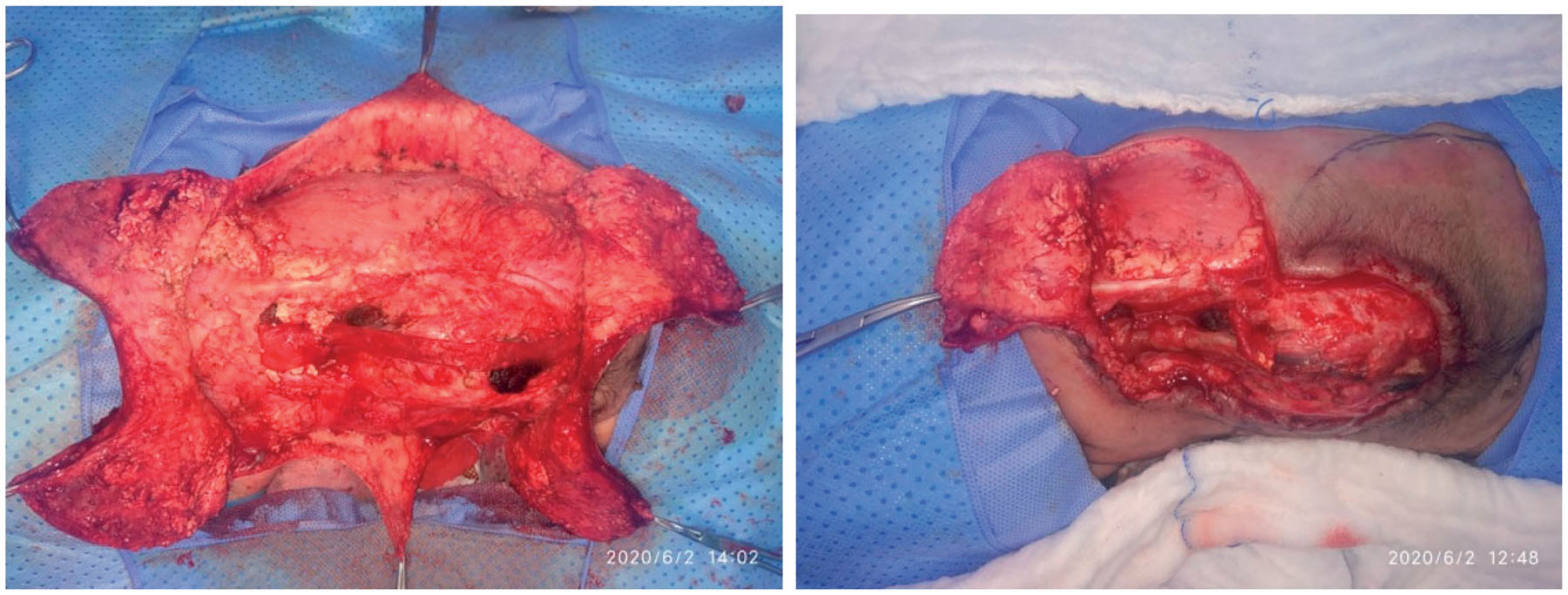
The rotation flaps are dissected below the muscular fascia level.

**Figure 3.**
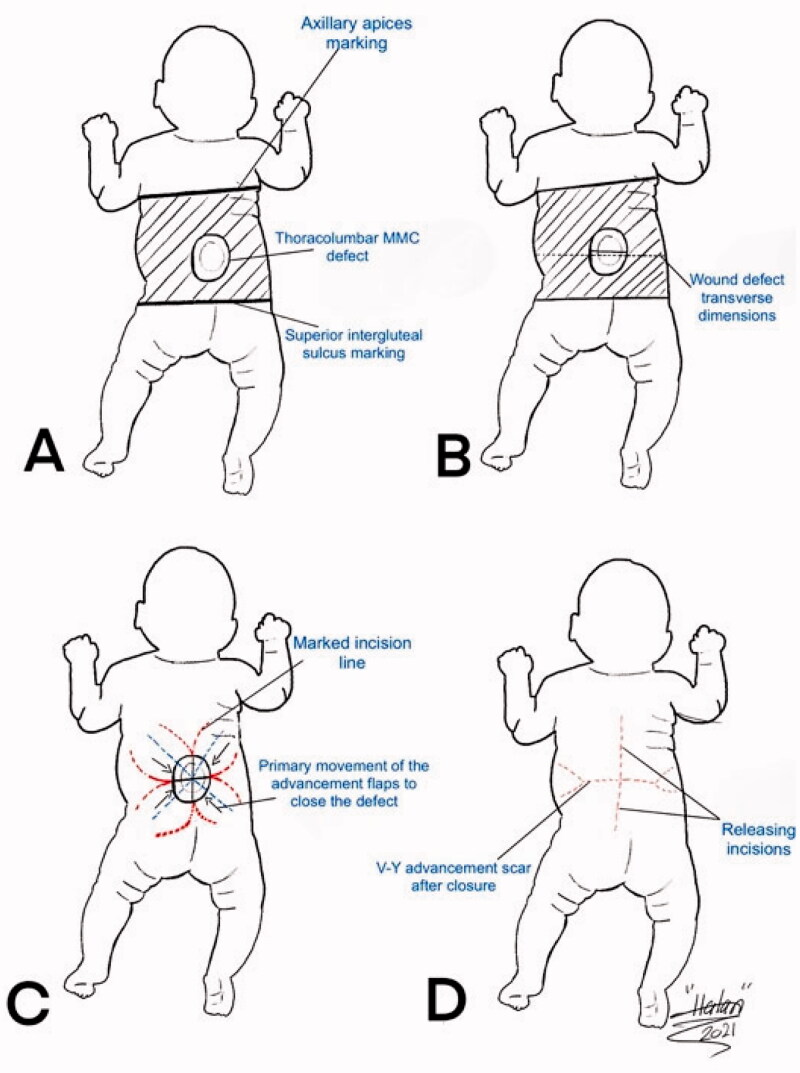
A drawing illustration explaining the marking of the V-Y rotation advancement flap. A. The axillary apices marking including most cranial point of the intergluteal sulcus, and posterior axillary lines forming a rectangular field shape. B. Drawing of the transverse dimensions on the wound defect. C. Quadruple flap drawing: the longest diameter of every V-Y rotation, and advancement flap dimension is one and a half more than half of each bisector of the defect. D. V-Y advancement scar after closure as well as the releasing incision scars at the top and bottom of the flaps.

**Figure 4.**
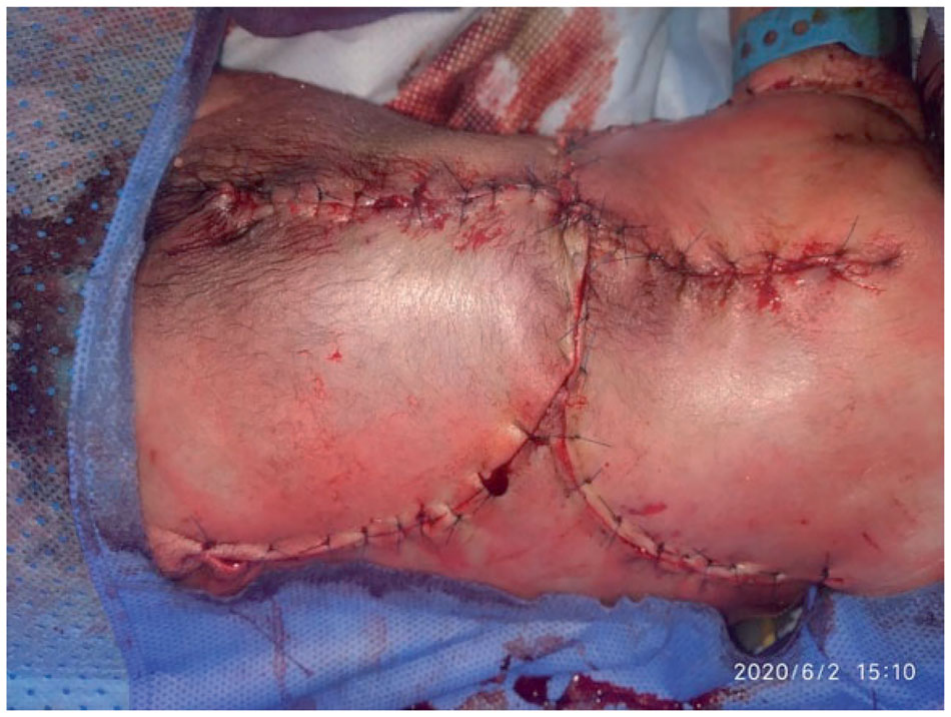
Appearance of the repair at the end of surgery.

The patient underwent joint operation performed by a neurosurgical and plastic surgical team under general anesthesia with oral intubation in a supine position and then turned to the prone position with the arms abducted by 90°. The neurosurgical team created a watertight dural sac from the MMC sac after the incision and separation of nerve fibers attached to it through microdissection under the surgical loupe. To reinforce the watertight closure, the neurosurgical team used 2-ml fibrin sealant glue (Tisseel kit) over the suture line to create an additional CSF leak barrier. The flap’s landmarks were marked, including the apices of the axilla bilaterally, the most superior point of the intergluteal sulcus, and the posterior axillary lines. The dissection limit to increase the flaps was demarcated by a line across the patient’s back through the most cranial point of the intergluteal sulcus and a second line connecting the axillary apices (Figure 2). The vertical and horizontal dimensions of the created surrounding rectangle and the defect to be covered by it were measured. The presence of kyphosis was considered to increase the extra flap length to ensure tension-free closure. The ratio of the maximum width of the defect to that of the back measured horizontally was 0.61. The rotation advancement axis for each of the four flaps was planned through an imaginary line bisecting each quadrant formed according to the defect’s horizontal and vertical axes, with the flap length of 1.5 × half the length of this line. The flap arms were drawn in an arc-shaped instead of a straight line exteriorly to obtain wider distal edges. As the flap drawing resembles the shape of a butterfly, it was called a butterfly flap. Adrenaline saline (1:100,000) was injected into the marked incisions. The thoracolumbar and latissimus dorsi (LD) fascia were incised and included in the flaps. Dissection was meticulously started from the medial side and continued until tension-free closure was achieved. Wider dissection was performed to protect the paraspinous perforators. Absolute hemostasis with bipolar therapy was achieved. Double-layer closure was performed with 4-0 polyglactin 910 and 5-0 poliglecaprone 25. To achieve tension-free closure over the center of the MMC, releasing incisions were made at the top and bottom of the flaps (Figure 3). After performing a layered closure using polydioxanone and poliglecaprone 25 sutures, the donor site was closed in a V–Y manner (Figure 4). No drain was needed.

### Postoperative care protocol

**Figure 5.**
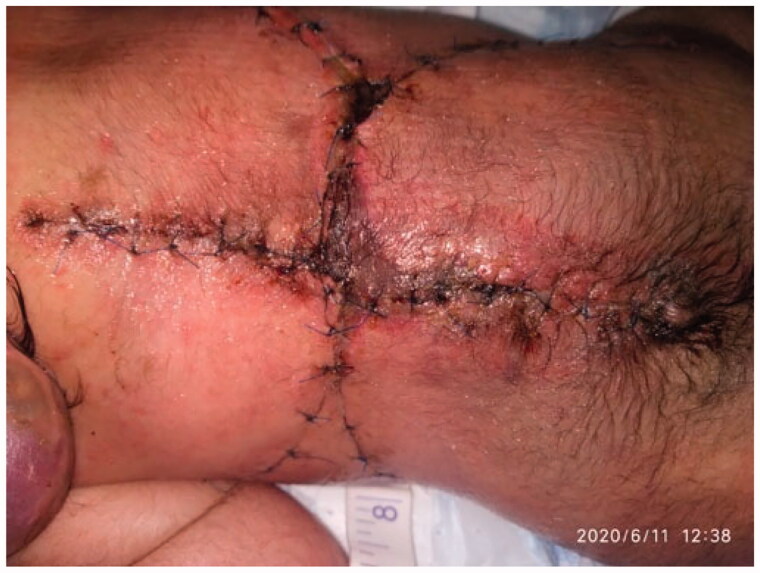
The repair 7 days postoperatively.

The patient was admitted to the NICU postoperatively. The baby was placed in prone position for 2 weeks postoperatively. The flap presented adequate perfusion postoperatively with a good capillary refill, without changes in color (cyanosis or pallor) (Figures 4 and 5). A 99% viability and late distal flap vascular compromise were observed on the left side; however, the healing was adequate. At 20 days old, the patient expired due to his comorbidities.

## Discussion

MMC is a congenital disorder of idiopathic cause due to closure failure of the posterior portion of the neural tube. The unusual growth of the vertebrae at the lamina and spinal apophysis level may involve one or more arcs, causing hernias that contain CSF and spinal cord meninges with a potential adhesion in the spinal cord or nerve roots. Multiple complications may occur in individuals with this defect. Therefore, early surgical intervention decreases the increasing number of the central nervous system infections and prevents the progression of neurological function deficit by protecting the neural tissue [7].

MMC defects can represent a challenging reconstructive challenge when the defect exceeds half of the back width. Approximately 25% of the defects cannot be managed with primary closure alone and require more advanced correction procedures. Most cases require primary closure with or without subcutaneous detachments [8]. Large or extensive soft-tissue defects require a more complicated approach. After MMC repair, reconstructions of large or extensive soft tissue defects have always been challenging for both plastic surgeons and neurosurgeons. A major issue is that the defect itself is large. Nevertheless, insufficient soft tissue may not be available to apply distance flap variations because the newborn has a restricted area in the back region [4,9]. Many various reconstructive options for large MMC defect closure have been mentioned in the literature. Therefore, we also conducted a literature review of all different methods used for large MMC closures (Table 1). In 1966, the first MMC surgery was initiated by applying the skin graft by Mustarde [10]. Previous studies using skin grafts for MMC found that some complications, such as graft ulceration, gibbus deformity, and severe kyphosis, and using split-thickness skin graft can lead to graft contraction. Because of these complications, the skin graft method does not offer sufficient tissue coverage over the repaired neural plate. Subsequently, musculocutaneous, muscle, and fasciocutaneous flaps have become more well known. In 1971, Desperez et al. [11] used a bipedicled thoracolumbar musculocutaneous flap to reconstruct a large MMC defect. One drawback of this method is that the donor area is usually closed with STSG due to the large donor area morbidity. McCraw et al. [12] have investigated the musculocutaneous flap LD method using double LD muscles. Both papers pointed out some issues, specifically, impossibility to reach for sacral MMC defects. The LD musculocutaneous flap does not have sufficient length for transfer to the sacral region and donor area grafting. They found that more than average blood loss was another disadvantage of their techniques. Regarding local flap methods, Cole et al. [13] applied purse-string suturing for small to medium MMC defects.

**Table 1.** Previously reported methods used in large MMC closure.

Number	First author	Type of study	Country of repair	Sample size	Operative technique	Complications
1.	Desprez [11]	Case series	United States of America	6	Vertical bipedicled	Not mentioned
2.	McCraw [12,14]	Case series	United States of America	5	Bipedicled latissimus dorsi musculocutaneos flaps	Not mentioned
3.	Moore [15]	Case series	United States of America	19	Bipedicled latissimus dorsi musculocutaneous flaps (with lateral relaxing incision)	Not mentioned
4.	Thomas [13]	Case series	Australia	4	Triangular advancement latissimus dorsi. Fasciocutaneous flaps (One or two)	Not mentioned
5.	Cruz [16]	Case series	United States of America	10 (Neonates)	Double Z rhomboid flap	Flap necrosis (*n* = 1)
6.	Ulusoy [4]	Case series	Turkey	10 (Neonates)	Bilateral modified V-Y advancement flap	Not mentioned
7.	El-sayed [17]	Case series	Egypt	16 (Neonates)	Proximally based fasciocutaneous flank flap	Superficial necrosis (*N* = 1) CSF leak (*n* = 1)
8.	Georgeana [18]	Case series	United Kingdom	3 (Children) 2 (Children)	Tissue expansion Advancement local flaps	Not mentioned

In this case report, we describe the use of a butterfly flap to cover a large MMC defect as this method was limited to either a latissimus turnover flap and skin grafting of the muscle or bipedicle fasciocutaneous flaps with skin grafting of donor sites. Given the possibility of functional outcomes in these patients due to neural tube defects, muscle usage is not ideal. Muscle sacrifice may decrease the strength during transfers as the patient grows and seeks independence. The LD is a reliable option, but these children pay the price of lifelong defect. This can be accepted as the MMC closure is considered lifesaving and prolonging the procedure in most cases. We now have a tool necessary to close these defects without sacrificing the muscle or skin grafting donor sites.

The first description of our butterfly flap technique was described by Kankaya et al. who reported a low rate of minor complications in their included patients postoperatively (18%) [5]. However, late small distal flap vascular compromise on the left side in our case occurred but was adequately healed with simple daily dressings and did not require further intervention. Conversely, closure using a butterfly flap was found to be a viable easy-to-implement alternative, reduces surgical time, minimizes blood loss, and does not require cutaneous grafting to cover the donor flap area.

Our technique has several significant advantages: it was a different flap alternative and was presented according to the anatomic localization and size, with a geometric flap design for all methods, no abnormal blood loss observed, the flap was easily transferred to the defect, and there was no need for skin grafting for both the flap and donor area; lastly it was a one-step surgery.

Therefore, we recommend the surgical management of large MCC defects using the technique presented here; however, further studies are needed to assess any potential unforeseen long-term complications to determine a valid and reliable visual analog scale for evaluating the management outcome in using the butterfly flap coverage for large MCC.

## Conclusion

Performing surgical management for severe large MMCs may be a challenging procedure. In our case report, we conclude that a butterfly advancement flap is an effective surgical technique for the closure of large MMC wound defects. The main advantages of using this technique are the short operative time, early healing, one-step surgery with excellent flap texture, and contour; however, more cases and longer follow-up are required.
